# Impact of a nutritional supplement during gestation and early childhood on child salivary cortisol, hair cortisol, and telomere length at 4–6 years of age: a follow-up of a randomized controlled trial

**DOI:** 10.1080/10253890.2020.1728528

**Published:** 2020-02-24

**Authors:** Brietta M. Oaks, Seth Adu-Afarwuah, Sika Kumordzie, Mark L. Laudenslager, Dana L. Smith, Jue Lin, Rebecca R. Young, Charles D. Arnold, Helena Bentil, Harriet Okronipa, Maku Ocansey, Kathryn G. Dewey

**Affiliations:** a Department of Nutrition and Food Sciences, University of Rhode Island, Kingston, RI, USA;; b Department of Nutrition and Food Science, University of Ghana, Legon, Ghana;; c Department of Nutrition, University of California, Davis, CA, USA;; d Department of Psychiatry, University of Colorado Anschutz Medical Campus, Aurora, CO, USA;; e Department of Biochemistry and Biophysics, University of California, San Francisco, CA, USA

**Keywords:** Cortisol, telomere length, pregnancy, nutrition, child

## Abstract

Dysregulation of the stress response can occur early in life and may be affected by nutrition. Our objective was to evaluate the long-term effect of nutritional supplementation during gestation and early childhood on child cortisol and buccal telomere length (a marker of cellular aging) at 4–6 years of age. We conducted a follow-up study of children born to women who participated in a nutritional supplementation trial in Ghana. In one group, a lipid-based nutrient supplement (LNS) was provided to women during gestation and the first 6 months postpartum and to their infants from age 6 to 18 months. The control groups received either iron and folic acid (IFA) during gestation or multiple micronutrients during gestation and the first 6 months postpartum, with no infant supplementation. At age 4–6 years, we measured hair cortisol, buccal telomere length, and salivary cortisol before and after a stressor. Salivary cortisol was available for 364 children across all three trial arms and hair cortisol and telomere length were available for a subset of children (*n* = 275 and 278, respectively) from the LNS and IFA groups. Telomere length, salivary cortisol, and hair cortisol did not differ by supplementation group. Overall, these findings suggest that nutritional supplementation given during gestation and early childhood does not have an effect on child stress response or chronic stress in children at 4–6 years.

**Trial registration:** ClinicalTrials.gov Identifier NCT00970866.

**Lay Summary**
- This study addressed a research gap about whether improved nutrition during pregnancy and early childhood impacts telomere length and cortisol in preschool children. There was no difference in child telomere length or cortisol between two trial arms of a nutritional supplementation trial that began during pregnancy. The research outcomes indicate lipid-based nutrient supplements, a relatively new form of supplementation, do not have an effect on markers of stress or cellular aging measured in later childhood.

## Introduction

Cortisol is a hormone released by the hypothalamic-pituitary-adrenal (HPA) axis in response to mental and physical stressors (Dickerson & Kemeny, 2004). Additionally, cortisol has a diurnal pattern in which cortisol concentrations typically peak 30–40 min after awakening and then decline throughout the day, with lowest concentrations in the evening (Hucklebridge, Hussain, Evans, & Clow, 2005). Previous studies have demonstrated that the regulation of both the stress response and diurnal pattern of cortisol can be impacted by a wide range of factors (Cohen et al., 2006; Danese & McEwen, 2012; Liu et al., 2017), including nutrition (Keenan et al., 2016; Oaks et al., 2016). Recent research suggests that permanent dysregulation of the HPA axis can occur during gestation and early childhood (Alexander et al., 2012). Whether nutrition during this critical time period can have a long-term impact on the HPA axis of the offspring is unknown.

Perceived stress and cortisol reactivity to stress are associated with shorter telomere length (Parks et al., 2009; Puterman et al., 2010), a biomarker of cellular aging (Collado, Blasco, & Serrano, 2007). Telomeres are structures at the end of chromosomes that become shorter with each cell division until reaching a limit at which point cell apoptosis or loss of cell function occurs. Shorter telomere length is associated with cardiovascular disease (Fitzpatrick et al., 2006), cancer (Ennour-Idrissi, Maunsell, & Diorio, 2017), and mortality (Cawthon, Smith, O'Brien, Sivatchenko, & Kerber, 2003). A meta-analysis of seven trials studying the impact of nutrition on telomere length reported no significant relationship; however, all trials have been conducted in adult populations and the meta-analysis noted strong heterogeneity among the studies in terms of type and duration of dietary intervention (Pérez et al., 2017).

We previously conducted a three-armed nutrition supplementation trial in Ghana, located in West Africa, to determine the effect of a LNS on maternal and child health outcomes when given during gestation, the first 6 months postpartum, and to the offspring from 6–18 months of age. In addition to greater birth weight and child growth in the LNS trial arm (Adu-Afarwuah et al., 2015), we found that among younger women, those provided with LNS during gestation had lower morning salivary cortisol in late gestation than those receiving iron and folic acid (IFA) capsules or multiple micronutrients (MMNs) capsules (Oaks et al., 2016). LNS and MMN had identical amounts of 18 micronutrients, but LNS also had four additional micronutrients and essential fatty acids, including the omega-3 fatty acid, alpha-linolenic acid, a necessary component for pathways that produce the anti-inflammatory response. As cortisol can cross the placenta, we now aim to determine if there was a lasting effect on their children.

The present study is part of a follow-up study conducted when the children were 4–6 years of age. We investigated whether the nutritional supplementation provided during gestation and early childhood had an impact on child stress response, hair cortisol (a measurement of cumulative cortisol), and buccal telomere length. We hypothesized that children in the LNS trial arm would have lower mean hair cortisol concentrations and a longer mean telomere length than children who received no supplementation and were born to mothers receiving IFA. We also hypothesized that the LNS children would have a better regulated stress response compared with children who received no supplementation and were born to mothers receiving IFA or MMN.

## Methods

### Participants, study design, and intervention

The International Lipid-Based Nutrients Supplement (iLiNS-DYAD) trial was a three-arm randomized controlled trial conducted in Ghana (Clinicaltrials.gov, NCT00970866). Details of the study design, randomization, and participants have been described previously (Adu-Afarwuah et al., 2015). Pregnant women ≤20 weeks gestation and ≥18 years of age were recruited from four prenatal clinics in the Yilo and Manya Krobo districts of the Eastern Region from December 2009 to December 2011. Exclusion criteria were: antenatal cards indicated HIV infection, asthma, epilepsy, tuberculosis, or any malignancy; known milk or peanut allergy; not residing in the area; intention to move within the next 2 years; unwillingness to receive fieldworkers in their home or take the study supplement; or participation in another trial. A total of 1320 enrolled women were randomized to receive either (1) LNS (during gestation and for 6 months postpartum), (2) MMNs (during gestation and for 6 months postpartum), or (3) IFA (gestation only). Children born in the LNS trial arm received LNS from 6 to 18 months of age while children in the MMN and IFA trial arms received no supplementation. The nutrient content for each of the supplements is presented in Table 1. After accounting for fetal loss and child deaths during the main trial, 1222 preschool children were eligible for the follow-up study conducted from January to December 2016. We attempted to contact all previous participants using contact information obtained during the main trial. All study protocols for the main trial and the follow-up study were approved by the institutional review board of the University of California, Davis, the Ethics Committee for the College of Basic and Applied Sciences at the University of Ghana, and the Ghana Health Service Ethical Review Committee.

**Table 1.** Nutrient supplementation per day.^a^

Nutrient	IFA	MMN	LNS-P&L	LNS-Child
Total energy (kcal)	0	0	118	118
Protein (g)	0	0	2.6	2.6
Fat (g)	0	0	10	9.6
Linoleic acid (g)	0	0	4.59	4.46
α-Linolenic acid (g)	0	0	0.59	0.58
Vitamin A (μg RE)	0	800	800	400
Vitamin C (mg)	0	100	100	30
Thiamin (mg)	0	2.8	2.8	0.3
Riboflavin (mg)	0	2.8	2.8	0.4
Niacin (mg)	0	36	36	4
Folic acid (μg)	400	400	400	80
Pantothenic acid (mg)	0	7	7	1.8
Vitamin B6 (mg)	0	3.8	3.8	0.3
Vitamin B12 (μg)	0	5.2	5.2	0.5
Vitamin D (µg)	0	10	10	5
Vitamin E (mg)	0	20	20	6
Vitamin K (μg)	0	45	45	30
Iron (mg)	60	20	20	6
Zinc (mg)	0	30	30	8
Copper (mg)	0	4	4	0.34
Calcium (mg)	0	0	280	280
Phosphorus (mg)	0	0	190	190
Potassium (mg)	0	0	200	200
Magnesium (mg)	0	0	65	40
Selenium (μg)	0	130	130	20
Iodine (μg)	0	250	250	90
Manganese (mg)	0	2.6	2.6	1.2

a IFA: maternal iron and folic acid; MMN: maternal multiple micronutrients; LNS-P&L: lipid-based nutrient supplements pregnancy and lactation formulation; LNS-Child: lipid-based nutrient supplements child formulation.

### Measurements

#### Cortisol stress response

To assess child cortisol stress response, we measured cortisol before and after a finger prick was performed on the child. We chose a finger prick as a stressor because the finger prick was already a part of our study protocol to collect blood for other study outcomes and had been supported as a stressor in previous research (Kertes, Kamin, Liu, Bhatt, & Kelly, 2018). We collected a total of four saliva samples: (1) during a home visit by a trained fieldworker, (2) upon arrival to the study testing center clinic, (3) 15 min after the finger prick, and (4) 30 min after the finger prick. We used polymer swabs specifically made for children less than 6 years of age (Salimetrics, LLC, State College, PA) to collect the saliva samples. The swab was inserted under the child’s tongue for 2 min and then placed in a storage tube. The swab collected during the home visit was stored in a cooler until the fieldworker returned to the study center. All swabs were stored in the refrigerator at the study center for up to 24 h. Swabs were then centrifuged and obtained saliva samples were stored at −20 °C until being shipped to the U.S. (Salimetrics, Carlsbad, CA) for lab analysis by high-sensitivity enzyme immunoassay, which can detect cortisol concentrations ranging from 0.193 to 82.8 nmol/L. The intra- and inter-assay coefficients of variability (CV) are 4.4% and 7.8%, respectively.

#### Hair cortisol

A small amount of hair (roughly the diameter of a pencil eraser and estimated to be at least 25 mg) was cut by the study nurse from the posterior vertex region close to the scalp. Typically, hair is estimated to grow 1 cm/month (Stalder et al., 2017); however, slower hair growth rate has been observed for persons of African descent (at an average rate of 0.80 cm/month) (Loussouarn, 2001). We therefore attempted to collect at least 1.6 cm of hair to estimate cortisol accumulation over the previous two months. Hair samples were wrapped in foil and stored at room temperature until shipped to the University of Colorado Anschutz Medical Center for analysis. Hair was ground, cortisol was extracted, and then measured by immunoassay (Salimetrics, LLC, State College, PA) according to previously published methods with average intra and inter-assay CVs 2.7% and 13.3%, respectively (Hoffman, Mazzoni, Wagner, Laudenslager, & Ross, 2016; Lehrer, Dubois, Maslowsky, Laudenslager, & Steinhardt, 2016).

#### Buccal telomere length

Buccal samples were collected by the study nurse using OraCollect-DNA swabs (DNA Genotek Inc, Ottawa, Canada). The swab was brushed up and down 10 times on the inside of each cheek, placed in a vial and immediately rotated by hand several times. To ensure sample homogeneity, the vial was heated in a wet bath incubator for 1 h at 50 °C before the sample was aliquoted into a cryovial. Buccal swabs were stored in the refrigerator for up to 24 h and stored at −20 °C until being shipped to University of California, San Francisco, CA, USA for lab analysis. DNA purification, using the Qiagen QiaAmp DNA mini kit, was carried out on saliva collected with DNA Genotek ORAcollect tubes. The telomere length measurement assay was adapted from the published original method by Cawthon (Cawthon, 2002; Lin et al., 2010) and represented a ratio of two qPCR reactions: Telomere over single copy gene (T/S). The samples measured for this study had an average T/S cv of 2.2 ± 1.6%. The telomere thermal cycling profile consisted of: cycling for T (telomic) PCR: denatured at 96 °C for 1 min, one cycle; denatured at 96 °C for one second, annealed/extended at 54 °C for 60 seconds, with fluorescence data collection, 30 cycles. Cycling for S (single copy gene) PCR: denatured at 96 °C for 1 min, one cycle; denatured at 95 °C for 15 seconds, annealed at 58 °C for one second, extended at 72 °C for 20 seconds, eight cycles; followed by denatured at 96 °C for one second, annealed at 58 °C for one second, extended at 72 °C for 20 seconds, held at 83 °C for five seconds with data collection, 35 cycles. The primers for the telomere PCR were tel1b (5′-CGGTTT(GTTTGG)5GTT-3′), used at a final concentration of 100 nM, and tel2b (5′-GGCTTG(CCTTAC)5CCT-3′), used at a final concentration of 900 nM. The primers for the single-copy gene (human beta-globin) PCR were hbg1 (5′-GCTTCTGACACAACTGTGTTCACTAGC-3′), used at a final concentration of 300 nM, and hbg2 (5′-CACCAACTTCATCCACGTTCACC-3′), used at a final concentration of 700 nM.

The final reaction mix contained 20 mM Tris–HCl, pH 8.4; 50 mM KCl; 200 mM each dNTP; 1% DMSO; 0.4x Syber Green I; 22 ng E. coli DNA; 0.4 Units of Platinum Taq DNA polymerase (Invitrogen Inc., Carlsbad, CA); approximately 10 ng of genomic DNA per 11 μl reaction. Tubes containing 26, 8.75, 2.9, 0.97, 0.324, and 0.108 ng of a reference DNA (pooled genomic DNA) were included in each PCR run so that the quantity of targeted templates in each research sample could be determined relative to the reference DNA sample by the standard curve method. The same reference DNA was used for all PCR runs. The samples for this study were handled in two plates (batches). The T/S ratio for each sample was measured twice. When the duplicate T/S value and the initial value varied by more than 7% for any sample, it was run a third time and the two closest values were reported. The repeat plate was a mixture from both study plates and was used to make batch adjustments. The CV for telomere length measurement in this study was 3.6%. All assays for the entire study were performed using the same lots of reagents. Lab personnel who performed the assays were provided with de-identified samples and were blind to all demographic and clinical data.

#### Covariates

Several variables were examined as potential covariates to reduce the within-group variance and to increase the precision of the estimate of the treatment effect in data analysis (Streiner, 2015). Potential covariates measured during the main trial included gestational age at enrollment, season at enrollment, parity, markers of inflammation at enrollment (C-reactive protein (CRP) and alpha (1)-acid glycoprotein (AGP)), pre-pregnancy body mass index (BMI), child sex, maternal age, maternal education, and maternal morning salivary cortisol at enrollment. Details of methods for these variables have been provided elsewhere (Oaks et al., 2016). Potential covariates measured during the follow-up study included child’s current age, season at time of sample collection, time between waking and time of saliva collection for saliva sample at clinic arrival, time between last food or drink and time of saliva collection for saliva sample at clinic arrival, and mood of child before saliva collection. Mood of the child was recorded by either the fieldworker or the study nurse as one of the following: happy (smiling), neutral (not upset, not happy), little upset (unhappy face, hesitant, shy), medium upset (some crying), or very upset (uncontrollable crying, screaming).

### Sample size calculation

The original trial enrolled 1320 pregnant women (∼440 per arm) to be able to detect an effect size (difference in means divided by the standard deviation) of ≥0.3 between groups in continuous outcomes with two-sided testing, an alpha of 0.05, power of 0.80, and assuming a 25% attrition rate while accounting for a known supplement mislabeling which resulted in mixed exposure. This follow-up study aimed to enroll all participants from the original sample who were still eligible to participate. A subsample of these follow-up participants was then selected for the current analyses.

For salivary cortisol, we conducted a three-group comparison and estimated a target subsample size of 480 children (160 per arm) to detect an effect size of ≥0.3 based on one-sided tests with an alpha of 0.05, power of 0.80, and accounting for 10–15% attrition. Buccal and hair sample collection was attempted for all children; however, due to limited funding for lab analysis we conducted a two-group comparison (LNS vs. IFA) which required a sample size of 278 (139 per arm) children to detect an effect size of ≥0.3 based on one-sided tests with an alpha of 0.05 and power of 0.80. Buccal samples for those two groups were selected for lab analysis using a randomized list created by our statistician. All available hair samples from the LNS and IFA groups were analyzed. These subsample sample size calculations were based on one-sided tests; however, two-sided tests were conducted in analysis. Consequently, our analyses are underpowered for detecting an effect size of 0.3; the detectable effect sizes are 0.35 for the salivary cortisol three-group comparison and 0.34 for the buccal and hair sample two-group comparisons.

### Statistical analysis

Prior to beginning any analyses, we made our statistical analysis plan publicly available by posting it on the iLiNS Project website (www.ilins.org). All models were checked to ensure that the residuals were normally distributed and the heteroscedasticity assumption was met through the residual vs. fit plot. Hair and salivary cortisol concentrations were consequently log transformed for analysis; no transformation was needed for telomere length. Outliers were identified by visually inspecting histograms and/or scatterplots of variables. In cases where extreme outliers were biologically plausible, sensitivity analysis was performed to determine whether the extreme outliers had undue influence on the results. High hair or salivary cortisol concentration was defined as a value above the median of the study population and short telomere length was defined as a value below the median of the study population. We calculated the area under the curve (AUC) for salivary cortisol concentration using the clinic arrival saliva sample and the 15 and 30 min post-finger prick saliva samples. AUC analyses were also run using the home saliva sample in place of the clinic arrival sample as a secondary analysis because of a concern that clinic arrival might itself be a stressor.

Primary analyses were two-sided and conducted as complete-case intention-to-treat. Secondarily, per protocol analyses were conducted limited to (a) women with ≥70% adherence to supplement during pregnancy and (b) women with ≥70% adherence during pregnancy and up to 6 months postpartum. Salivary cortisol concentration was compared across three groups (IFA, MMN, LNS) and hair cortisol concentration and telomere length were compared between two groups (IFA vs. LNS). Both unadjusted and adjusted analyses were completed, because guidelines for best statistical practices support the use of covariates in analyses of randomized controlled trials (Streiner, 2015). Outcomes were first assessed in unadjusted models controlling only for study design covariates and then in models adjusting for covariates associated (*p* < .10) with the outcome. For continuous outcome variables, the difference in means between groups was tested with ANCOVA using a statistical significance of *p* < .05. For dichotomous outcome variables, odds ratios between groups were examined using logistic regression with statistical significance at *p* < .05. Post hoc pairwise comparisons of the three intervention groups were performed only if the global ANCOVA *F*-test was significant in relation to either continuous or dichotomous salivary cortisol concentrations.

All models controlled for the child’s age since not all children were assessed at the same age during the follow-up. Additionally, time between waking and time of saliva collection for the baseline saliva sample, and time between last food or drink and time of saliva collection for the baseline saliva sample were also included in all salivary cortisol models, as these are established factors that affect salivary cortisol concentrations. Variables examined as potential adjustment covariates included child sex, maternal age, maternal education, gestational age at enrollment, parity, maternal AGP at enrollment, maternal CRP at enrollment, pre-pregnancy BMI, season at enrollment, maternal morning salivary cortisol at enrollment (salivary cortisol analyses only), child mood (salivary cortisol analyses only), and season at time of child saliva collection (salivary cortisol analyses only). We examined potential interaction of supplementation group with pre-specified variables, which included child sex, maternal cortisol at enrollment, maternal AGP at enrollment, maternal CRP at enrollment, maternal age, maternal parity (nulliparous vs. parous), and season at time of sample collection. These variables were selected based on previous research suggesting that these factors may act as effect modifiers (Adu-Afarwuah et al., 2015; Bosquet Enlow et al., 2019; Oaks et al., 2016; Toe, Bouckaert, & De Beuf, 2015). Maternal BMI was examined as an effect modifier in exploratory analyses based on other recent analyses completed by our research group (Kumordzie et al., 2019). We performed sub-group analyses for significant interactions (*p* < .1). To address the issue of multiple hypothesis testing, any interactions identified as significant in the main analysis were also examined after performing a Bonferroni correction (Cabin & Mitchell, 2000). All analyses were completed using SAS 9.4 (SAS Institute, Cary, NC).

## Results

### Salivary cortisol

**Figure 1.**
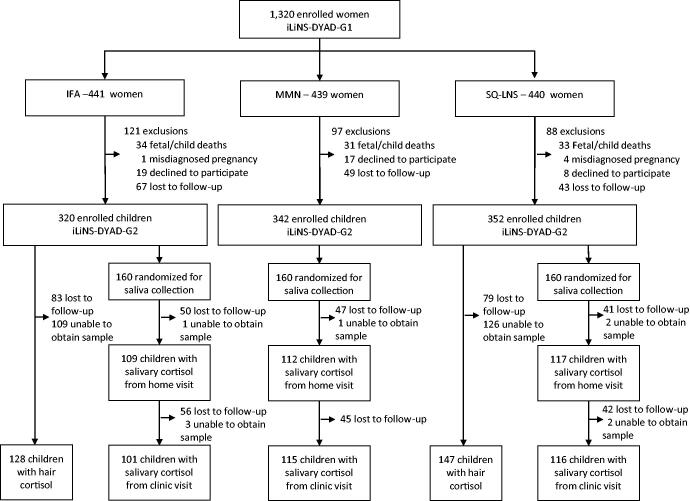
Study participant flow diagram for iLiNS-DYAD-G2 child cortisol analysis.

From the 1222 preschool children that were eligible, a total of 1014 children (83%) were enrolled in the iLiNS-DYAD follow-up study in Ghana. From the 480 children randomized for saliva collection, we collected saliva from 338 (70%) children at the home visit and from 332 (69%) children at the clinic visit. Of the 148 children who did not provide a saliva sample at the clinic visit, five attended the clinic visit but were unable to provide a sample and the remaining were lost to follow up (i.e. did not attend the clinic visit) (Figure 1). Compared with children without a saliva sample obtained at the clinic visit, children with a saliva sample were similar in age; had similar hair cortisol concentration; and had mothers that were similar in education level, salivary cortisol, and BMI at trial enrollment but were on average slightly older (mean age: 27.2 vs. 26.6 years, *p*=.08). Baseline characteristics were similar across intervention groups and characteristics of study participants by intervention group are presented in Table 2.

**Table 2.** Characteristics of study participants by intervention group^a^.

Characteristic	IFA	MMN	LNS
	*n* = 210^b^	*n* = 121^b^	*n* = 223^b^
Birth weight, kg	2.94 ± 0.44	2.96 ± 0.46	3.04 ± 0.40
Male, %	97 (46%)	50 (42%)	107 (48%)
Preterm, %	14 (7%)	11 (9%)	17 (8%)
Length-for-age *z*-score (LAZ) at birth	−0.72 ± 1.02	−0.59 ± 1.05	−0.54 ± 0.93
Gestational age at enrollment, weeks	16.2 ± 3.3	15.7 ± 3.1	16.0 ± 3.5
Maternal age at enrollment, years	26.8 ± 5.5	27.4 ± 5.6	27.2 ± 5.5
Primiparous, %	67 (32%)	32 (26%)	74 (33%)
Maternal cortisol at enrollment, nmol/L	5.23 ± 2.86	4.75 ± 2.68	4.58 ± 2.44
Maternal cortisol at 36 weeks gestation, nmol/L	8.02 ± 3.30	7.49 ± 3.36	7.80 ± 3.13

IFA: maternal iron and folic acid; MMN: maternal multiple micronutrients; LNS: maternal and child lipid-based nutrient supplements.

a Values are mean ± SD or *n* (%), as indicated.

b Missing data: birth weight, LAZ, and preterm (IFA: *n* = 8, MMN: *n* = 4, LNS: *n* = 5); cortisol at enrollment (IFA: *n* = 57, MMN: *n* = 32, LNS: *n* = 75); cortisol at 36 weeks gestation (IFA: *n* = 89, MMN: *n* = 50, LNS: *n* = 96).

**Figure 2.**
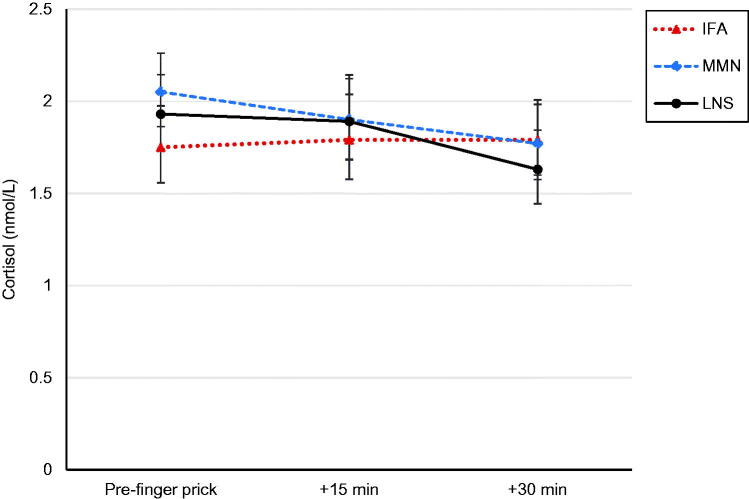
Change in salivary cortisol among children 4–6 years of age before and after a finger prick, by supplement group. Data represent geometric mean cortisol (error bars 95% CI) controlling for child age, time since awakening, and time since eating. IFA: maternal iron and folic acid group; MMN: maternal multiple micronutrients group; LNS: maternal and child lipid-based nutrient supplements group.

Geometric mean (95% CI) salivary cortisol concentration at home was 4.00 (3.68, 4.35) nmol/L and did not differ by group (*p*=.75). The majority of home visits were conducted early in the morning before the child went to school, with fieldworkers typically collecting saliva from the child before 6:30 am. Clinic visits were conducted between the hours of 8 am and 12 pm and geometric mean salivary cortisol concentration at clinic arrival was 1.92 (1.80, 2.04) nmol/L. Compared with the clinic arrival salivary cortisol concentration, 55% of the children had either a decrease or no change in cortisol 15 min post-finger prick. Geometric mean salivary cortisol concentrations measured 15 and 30 min post-finger prick were 1.87 (1.74, 2.00) and 1.73 (1.62, 1.85), respectively. Due to relatively little difference in mean cortisol concentrations across the three saliva samples collected at the clinic visit, AUC analyses are not presented. Salivary cortisol concentrations did not differ significantly among the three nutrition supplementation groups (Figure 2). Per protocol analysis results were similar to the intention-to-treat results and are not presented. There were no significant interactions with supplementation for any of the examined effect modifiers when analyzed in separate models.

### Hair cortisol

Hair sample collection was attempted on all children and lab analysis was limited to the IFA and LNS groups. Of the 672 children in either the IFA or LNS groups, hair samples were successfully collected from only 275 children (41%). Hair samples were not collected from the other children because of either loss-to-follow-up (*n* = 162) or inability to obtain a hair sample from the child at the clinic visit (*n* = 235), primarily due to hair being too short (Figure 1). Compared with children without a hair sample, children with a hair sample were similar in age; had mothers that were similar in age, education level, salivary cortisol, and BMI at trial enrollment; had a higher mean salivary cortisol concentration at clinic arrival (2.34 vs. 2.01, *p*=.01) but similar salivary cortisol at other time points; and were slightly more likely to be female (55% vs. 50%, *p*=.11).

Before hair samples were collected at the clinic visit, mothers were asked about the frequency of hair washing for the child. All mothers reported washing their child’s hair within the past 48 h and 93% of mothers reported that the child’s hair was typically washed twice per day, with no significant variation in hair washing frequency between the IFA and LNS groups. Hair cortisol concentration did not significantly differ between the IFA and LNS groups (Table 3). Per protocol analysis results were similar to the intention-to-treat results and are not presented. There were no significant interactions with supplementation for any of the examined effect modifiers when analyzed in separate models.

**Table 3.** Hair cortisol and telomere length by intervention group^a^.

	IFA^b^	LNS^b^	*p*
Hair cortisol, *n*	128	139	
Hair cortisol, pg/mg	5.37 (4.61, 6.28)	4.95 (4.40, 5.57)	.29
Telomere length, *n*	147	139	
Telomere length, T/S ratio	1.75 (1.67, 1.83)	1.71 (1.63, 1.79)	.69

a Values are means ad 95% CI, adjusted for child age.

b IFA: maternal iron and folic acid; LNS: maternal and child lipid-based nutrient supplements.

### Buccal telomere length

Buccal sample collection was attempted on all children and buccal swabs were collected from 819 children (81%). Lab analysis was limited to children from the IFA and LNS groups. For the subsample selection for buccal swab analyses, all children who provided a hair sample were included; however, several children from the hair cortisol subsample were missing a buccal swab and additional children were randomized to obtain our target sample size of 278 children (LNS: *n* = 139, IFA: *n* = 139). Mean buccal telomere length among children from the LNS group did not differ significantly from the IFA group (Table 3). Results were similar and the difference between groups remained non-significant in adjusted analyses and per protocol analyses. Of the eight examined potential effect modifiers, there was an interaction with maternal age (*p*-for-interaction=.06). Among children born to older mothers, telomere length was longer for children from the LNS group compared with the IFA group. Among children born to younger mothers, telomere length was shorter for children from the LNS group compared with the IFA group. However, the interaction did not remain significant after performing a Bonferroni correction, nor did the intervention groups differ significantly when compared within each of two maternal age categories (above and below the median age at enrollment, 26 years). No other interactions with supplementation were significant when analyzed in separate models.

## Discussion

Our previous research from the main trial in Ghana showed that among younger women, late pregnancy morning salivary cortisol concentration was lower among those receiving LNS compared with those receiving IFA or MMN (Oaks et al., 2016). In this follow-up study, we examined the hypothesis that provision of LNS during gestation and early childhood would alter offspring salivary and hair cortisol concentrations and buccal telomere length at 4–6 years of age. Buccal telomere length, hair cortisol concentrations, and salivary cortisol concentrations before and after a finger prick were similar across supplementation groups, thus our results do not support our primary hypothesis. We did find evidence of an interaction between the nutrition supplement and maternal age: among children born to older mothers, those in the LNS group had longer telomere length than those in the IFA group, with the opposite seen among children born to younger mothers. However, it is possible that this finding was due to chance, as the interaction did not remain significant after correcting for multiple hypothesis testing.

To our knowledge, this is the first study to examine the long-term effect of a nutrition supplement given during both gestation and early childhood on either child telomere length or cortisol concentration. Previous studies have shown that nutrition supplementation during gestation modulates the offspring salivary cortisol response to a stressor in both animals (Grissom, George, & Reyes, 2017) and humans (Keenan et al., 2016). In the present study, salivary cortisol concentration generally did not exhibit a stress response to the finger prick. Previous studies have reported that 4–6 year olds are one of the more challenging groups in which to elicit the stress response (Gunnar, Talge, & Herrera, 2009). Future studies would benefit from exploring other stressors.

In terms of child telomere length, our results are consistent with a prenatal omega-3 supplementation trial that showed no effect on child telomere length at 12 years of age (See et al., 2016) and a study of the Dutch famine birth cohort in which undernutrition during gestation was not associated with offspring telomere length at 68 years of age (de Rooij et al., 2015). However, two other cohort studies have reported that higher serum folate and vitamin D concentrations during pregnancy are associated with longer telomere length in newborns (Entringer et al., 2015; Kim et al., 2018). In our study, all three groups received folate so it is possible that folate has an impact on telomere length that we could not examine in this study. However, only the LNS and MMN groups received vitamin D as part of the assigned supplement and we did not find a difference in telomere length between the IFA and LNS groups, so our study does not support a focus on maternal vitamin D supplementation for impacting offspring telomere length in this study setting. It is possible that the nutritional status of the mother during gestation has an effect on newborn telomere length that then is not evident at older ages, although research is still limited in this area and further investigation is needed.

There are several biological mechanisms that could underlie effects of a maternal or early childhood nutritional supplement on child cortisol and telomere length. Omega-3 fatty acids can facilitate the anti-inflammatory response. As inflammation is associated with both higher cortisol (Silverman & Sternberg, 2012) and shorter telomere length (Wong, Vivo, Lin, Fang, & Christiani, 2014), it is possible that omega-3 supplementation may lead to lower cortisol concentrations and longer telomeres. This is particularly relevant during pregnancy, which is a state of chronic inflammation. Folate acts as a methyl donor and is necessary for fetal DNA synthesis and cell proliferation. Folate deficiency can lead to a compromised DNA structure that may affect the telomere sequence (Entringer et al., 2015). Vitamin D can upregulate telomerase activity, an essential enzyme for telomere maintenance. Additionally, Vitamin D promotes the expression of Klotho, a protein associated with cellular aging. An *in vitro* study using human umbilical cells demonstrated that Klotho deficiency induces telomere shortening (Buendía et al., 2015).

Our study has several strengths and limitations worth noting. We had relatively low loss-to-follow-up, enrolling approximately 80% of children from the main trial in the follow-up study. While any loss-to-follow-up can contribute to attrition bias, the likelihood of attrition bias for this study is low. We found baseline characteristics to be similar between those with and without a saliva sample at 4–6 years of age aside from a slight difference in maternal age. We measured cortisol by two different indicators, hair and saliva, and included measurement of variables that often affect these variables, such as hair washing and time of awakening. Limitations include difficulty in collecting hair, which may affect the generalizability of our results, as children with shorter hair may differ from children with longer hair (e.g. hair length could possibly be associated with sex of the child, economic status, maternal care, etc.). However, we compared baseline characteristics of children who did and did not have a hair sample and found little difference between these two groups. Our sample size calculations were based on our primary hypothesis and one-sided tests, so it is possible that the study, including analysis of interactions, was underpowered. We have previously reported that at 18 months of age, 43% of the children in this trial were anemic (Adu-Afarwuah et al., 2019) and at 4–6 years of age, there were relatively low rates of stunting (6.3%), underweight (5.9%), and overweight (2.9%) (Kumordzie et al., 2019). Our results may not be generalizable to populations that differ in these and other indicators of health status. Due to the study setting we were not able to collect cortisol at set time points, which increases variance in the cortisol measurement because cortisol has a diurnal rhythm. To address this issue, we recorded the time of collection for each participant and included it as a covariate in our analyses. We also were not able to assess the cortisol awakening response or evening cortisol, which limits comparability to other studies. Our stress test was conducted in the morning and may have been influenced by the diurnal rhythm of cortisol, as cortisol decreases during morning hours after a sharp increase after awakening. However, children had typically been awake for several hours and the mean cortisol at clinic arrival was notably lower than the morning cortisol collected at home.

## Conclusions

Research on the long-term effects of improving nutrition during both gestation and childhood is needed to determine the full impact of nutrition interventions when focused on the first 1000 days (gestation and the first 2 years), providing critical information to maternal and child nutrition programs. This study has contributed toward addressing this research gap by examining child cortisol and telomere length at 4–6 years of age among children from a prenatal and early childhood nutrition supplementation trial. Our results suggest that nutrition supplementation had little to no effect on these markers of stress and cellular aging at this time point in the child’s life. Further research exploring other indicators of health and at other time points for the offspring are needed to fully understand the impact of prenatal and early childhood nutrition.

## Data Availability

The data that support the findings of this study are available from the corresponding author, BMO, upon reasonable request. The data are not publicly available due to their containing information that could compromise the privacy of research participants.

## Names for PubMed indexing

Oaks, Adu-Afarwuah, Kumordzie, Laudenslager, Lin, Smith, Young, Arnold, Bentil, Okronipa, Ocansey, Dewey.
